# Atrial Fibrillation and Mortality after Gastrointestinal Surgery: Insights from a Systematic Review and Meta-Analysis

**DOI:** 10.3390/jpm14060571

**Published:** 2024-05-27

**Authors:** Alexandru Cosmin Palcău, Liviu Ionuț Șerbănoiu, Daniel Ion, Dan Nicolae Păduraru, Alexandra Bolocan, Florentina Mușat, Octavian Andronic, Ștefan-Sebastian Busnatu, Adriana Mihaela Iliesiu

**Affiliations:** 1 “Carol Davila” University of Medicine and Pharmacy, 050474 Bucharest, Romania; alexandru-cosmin.palcau@drd.umfcd.ro (A.C.P.); daniel.ion@umfcd.ro (D.I.); dan.paduraru@umfcd.ro (D.N.P.); alexandra.bolocan@umfcd.ro (A.B.); florentina.musat@drd.umfcd.ro (F.M.); octavian.andronic@umfcd.ro (O.A.); stefan.busnatu@umfcd.ro (Ș.-S.B.); adriana.iliesiu@umfcd.ro (A.M.I.); 2 General Surgery Department, University Emergency Hospital of Bucharest, 050098 Bucharest, Romania; 3 Department of Cardiology, Emergency Hospital “Bagdasar-Arseni”, 050474 Bucharest, Romania; 4 Department of Cardiology, “TH. Burghele” Hospital, 050659 Bucharest, Romania

**Keywords:** atrial fibrillation (AF), mortality, gastrointestinal (GI) surgery, postoperative complications

## Abstract

Background: Heart failure, stroke and death are major dangers associated with atrial fibrillation (AF), a common abnormal heart rhythm. Having a gastrointestinal (GI) procedure puts patients at risk for developing AF, especially after large abdominal surgery. Although earlier research has shown a possible connection between postoperative AF and higher mortality, the exact nature of this interaction is yet uncertain. Methods: To investigate the relationship between AF and death after GI procedures, this research carried out a thorough meta-analysis and systematic review of randomized controlled studies or clinical trials. Finding relevant randomized controlled trials (RCTs) required a comprehensive search across many databases. Studies involving GI surgery patients with postoperative AF and mortality outcomes were the main focus of the inclusion criteria. We followed PRISMA and Cochrane Collaboration protocols for data extraction and quality assessment, respectively. Results: After GI surgery, there was no statistically significant difference in mortality between the AF and non-AF groups, according to an analysis of the available trials (*p* = 0.97). The mortality odds ratio (OR) was 1.03 (95% CI [0.24, 4.41]), suggesting that there was no significant correlation. Nevertheless, there was significant heterogeneity throughout the trials, which calls for careful interpretation. Conclusion: Despite the lack of a significant link between AF and death after GI surgery in our study, contradictory data from other research highlight the intricacy of this relationship. Discrepancies may arise from variations in patient demographics, research methodology and procedural problems. These results emphasize the necessity for additional extensive and varied studies to fully clarify the role of AF in postoperative mortality in relation to GI procedures. Comprehending the subtleties of this correlation might enhance future patient outcomes and contribute to evidence-based therapeutic decision making.

## 1. Introduction

Gastrointestinal (GI) procedures are essential tools in modern medicine, but they are not without risks. A notable consequence is atrial fibrillation (AF), an irregular and typically rapid heart rhythm that affects an estimated 2.47% of males and 1.56% of females in the UK and that is connected to an increased risk of stroke, heart failure and death [1,2,3,4]. GI surgeries, especially major abdominal procedures, have a greater incidence of postoperative AF [5].

Recent epidemiological research estimates that 33.5 million individuals worldwide have AF, with prevalence rising to 10% in those over 80 [6]. During surgery, an increased risk of myocardial infarction and stroke, two adverse cardiovascular events that boost morbidity and mortality rates, have been associated with AF [7]. Furthermore, the occurrence of AF during GI surgeries is related to a considerable increase in mortality. A study found out that individuals with AF after gastrointestinal surgery had a 2.4 times greater risk of death compared to those without AF [8].

AF happens between 20.8% and 27% of the time after heart surgery. It is well known to be linked to both short-term and long-term death probability [9,10]. However, not much research has been conducted on AF after general surgery, especially abdominal surgery. A new study found that between 12 and 15% of people who had stomach surgery had AF [11]. New-onset AF can be caused by other heart or lung problems and can happen during issues like “anastomotic leak” or pelvic collection [11,12]. Some people have AF without any obvious underlying disease. Having a longer hospital stay is linked to more severe new-onset AF [12] and a higher risk of stroke within 30 days [13].

This systematic review and meta-analytic study looked into the connection between gastrointestinal problems and AF after abdominal surgery. The study’s goal was to find out how AF affects the death rate after stomach or GI surgeries, and the mortality considered was all-cause mortality following postoperative atrial fibrillation. This study looked at only randomized clinical trials and the results of those trials in order to find important factors for better evidence-based decisions.

## 2. Methods

**Figure 1:**
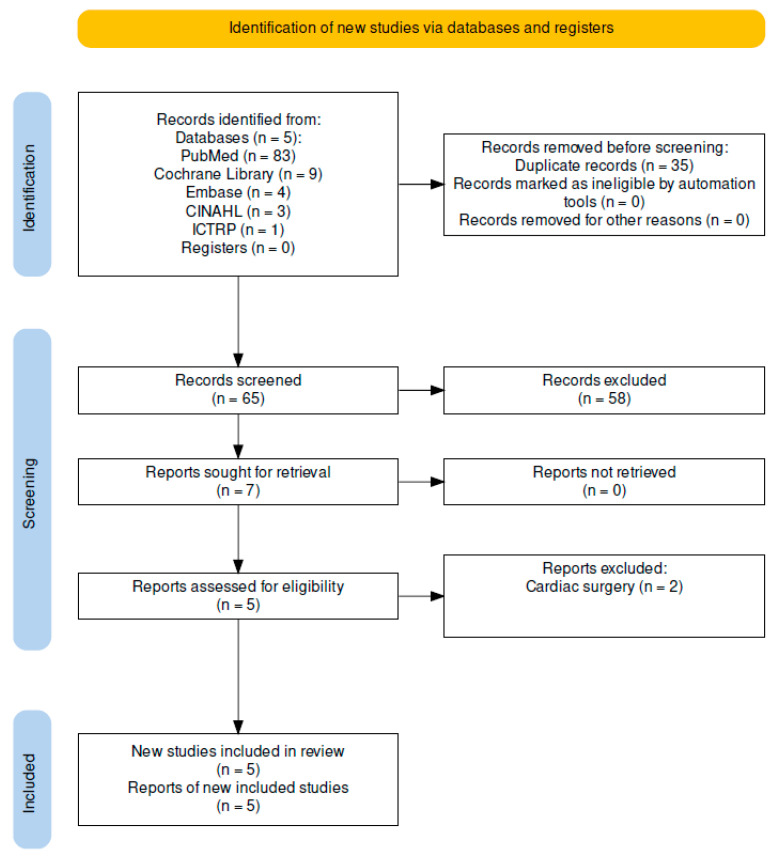
PRISMA Flow Diagram made in Revman.

Throughout our investigation, we adhered to the Preferred Reporting Items for Systematic Reviews and Meta-Analyses (PRISMA) guidelines. The PRISMA 2020 checklist is shown in Figure 1. These guidelines provide a uniform framework that guarantees a methodical way of disclosing collected data and, therefore, the outcomes of systematic reviews and meta-analyses. We registered our protocol on PROSPERO, and the registration ID is as follows: CRD42024540505.

### 2.1. Literature Database Search

First, we carried out an extensive search across five databases, namely PubMed, Embase, CINAHL, ICTRP and Cochrane Library, through employing specific keywords and Boolean operators, including ((“digestive system surgical procedures”[MeSH Terms] OR (“digestive”[All Fields] AND “system”[All Fields] AND “surgical”[All Fields] AND “procedures”[All Fields]) OR “digestive system surgical procedures”[All Fields] OR (“gastrointestinal”[All Fields] AND “surgery”[All Fields]) OR “gastrointestinal surgery”[All Fields] OR (“GI”[All Fields] AND (“surgery”[MeSH Subheading] OR “surgery”[All Fields] OR “surgical procedures, operative”[MeSH Terms] OR (“surgical”[All Fields] AND “procedures”[All Fields] AND “operative”[All Fields]) OR “operative surgical procedures”[All Fields] OR “general surgery”[MeSH Terms] OR (“general”[All Fields] AND “surgery”[All Fields]) OR “general surgery”[All Fields] OR “surgery s”[All Fields] OR “surgerys”[All Fields] OR “surgeries”[All Fields]))) AND (“atrial fibrillation”[MeSH Terms] OR (“atrial”[All Fields] AND “fibrillation”[All Fields]) OR “atrial fibrillation”[All Fields]) AND (“mortality”[MeSH Terms] OR “mortality”[All Fields] OR “mortalities”[All Fields] OR “mortality”[MeSH Subheading])) AND (clinicaltrial[Filter] OR randomizedcontrolledtrial[Filter]) since the inception of the databases. No filters were applied for the time of publication.

### 2.2. Inclusion Criteria and Study Selection

We developed appropriate inclusion criteria and used the Population, Intervention, Comparator group and Outcomes (PICO) framework to assess the full-text studies. These criteria included the following elements: (1) randomized controlled trials (RCTs); (2) studies published in English; (3) studies with no time restriction on publication; (4) patients undergoing any type of gastrointestinal surgery, which are the study’s target population; and (5) patients who experienced AF as a complication and later died as a result of it. In order to maintain the rigor of our methodology procedure, our study also established clear exclusion criteria. These included the following: (1) studies that were not RCTs; (2) studies that were not in English; (3) studies that included patients younger than 18 years of age; (4) studies that addressed mental disorders; and (5) studies that did not include any control data. 

For the experimental group in our study, the following criteria needed to be met: patients who underwent GI surgery and experienced AF. Patients who underwent GI surgery but did not develop postoperative AF were considered for the control group. 

### 2.3. Extracting Data and Evaluating Quality

In order to discover and analyze relevant studies and provide reliable and accurate results, an orderly approach was taken. The items were moved to the EndNote Reference Library after a thorough search. Duplications were removed in order to ensure that the study included only unique findings. Research studies that met the set standards for inclusion were carefully reviewed using their full text. There were two steps to the review process. First, the titles and abstracts of each study were looked at to see how relevant they were to the research question. Studies that did not match the selection criteria were eliminated at this point. Second, a full-text reading and discussion were performed on the leftover articles to ensure they met the selection criteria and to spot any inconsistencies (Table 1). An Excel spreadsheet was used to extract and assemble the trials’ final findings. Each and every piece of data was carefully documented and stored for further research. Our goal was to assess the mortality rate in the experimental and control groups after AF that happened either during or after surgery.

**Figure 2:**
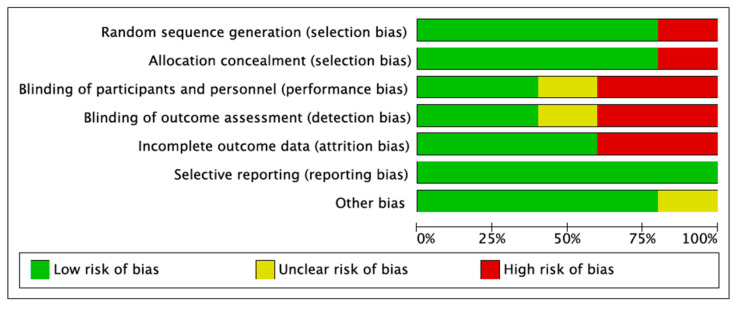
Risk of bias graph.

The risk of bias approach for randomized controlled trials, developed by the Cochrane Collaboration, was used to assess the quality of each research paper. This metric was used to evaluate each study’s methodology, bias risk and internal validity (Figure 2).

**Figure 3:**
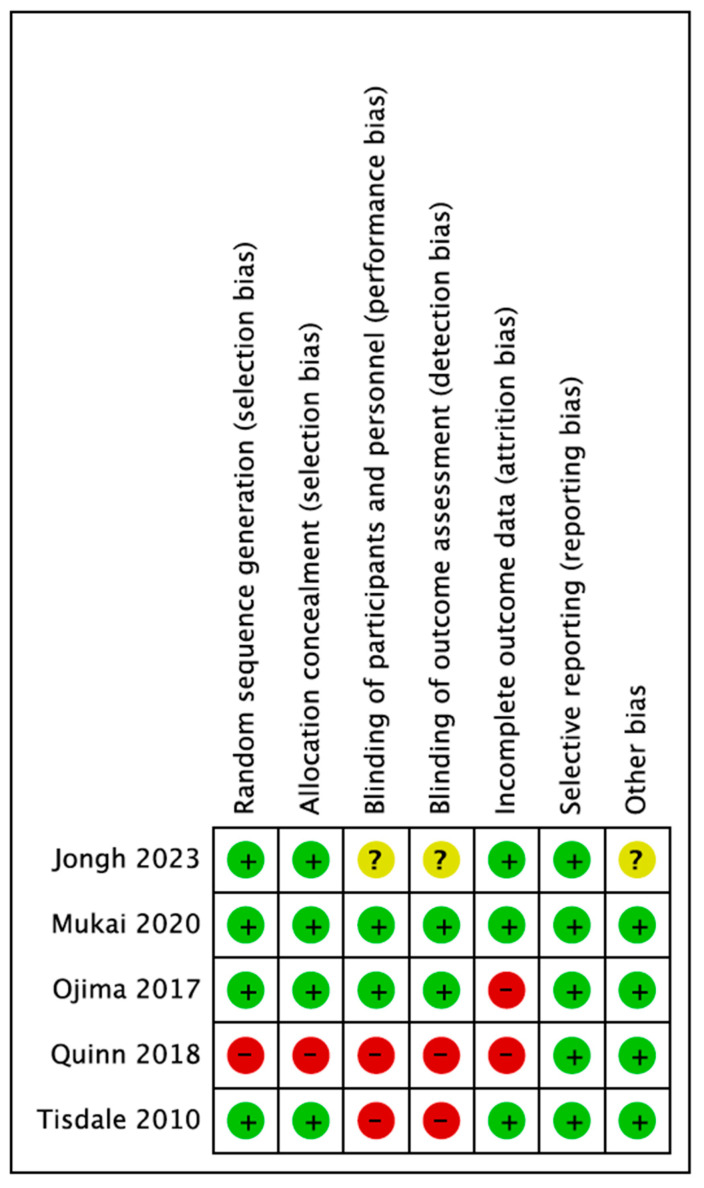
Risk of bias summary [14,15,16,17,18].

When calculating the bias, a number of factors were taken into account, including reporting, blinding, attrition, randomization and allocation concealment. The findings for each research paper and each location are summarized using a color-coded approach (low, high or indeterminate risk of bias) in the graph and chart. This technique made it easier to identify and eliminate any biases that would have jeopardized the accuracy and practicality of the findings (Figure 3).

### 2.4. Meta-Analysis

Two analyses were carried out using Meta Analysis Review Manager (RevMan) software (Version 5.4; Copenhagen: The Cochrane Collaboration, 2020) based on data that were retrieved from each study. In the first meta-analysis, the mean prevalence of atrial fibrillation was compared using a random effects model for the experimental and control groups. In the second analysis, we analyzed data regarding mortality due to postoperative atrial fibrillation in the experimental and control groups. The association was visualized using forest plots for each outcome. Moreover, any biases were investigated by creating a funnel plot using the RevMan program in order to confirm the reliability and validity of our results.

## 3. Results 

### 3.1. Study Characteristics

From the initial total number of studies identified (n = 100), 35 duplicates were identified and removed. A total of 58 studies were excluded from the remaining 65 studies, as they were books, documents, reviews, systematic reviews, meta-analyses, letters to the editor, only abstracts or not originally English-written. A total of 5 studies adhered to our inclusion criteria from the remaining 7 studies and were thus included, as demonstrated by Figure 1, which presents the selection process in accordance with PRISMA standards.

### 3.2. Mortality

**Figure 4:**
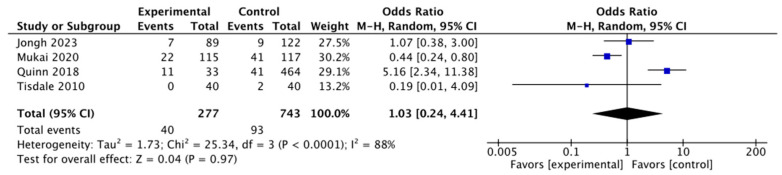
Mortality forest plot [14,15,17,18].

Data from four studies—Jongh 2023 [14], Mukai 2020 [15], Quinn 2018 [18] and Tisdale 2010 [17]—were analyzed for this result. The mortality considered was all-cause mortality following postoperative atrial fibrillation. In our forest plot analysis, each row represents a single study, and the columns show the numbers for the Experimental Events/Total Participants and the Control Events/Total Participants (Figure 4). The odds ratio, or OR, was used in this research to assess the relationship between the exposure (AF) and the result (mortality). Using a random effects model and the Mantel–Haenszel technique, the OR was determined for each study. The range of the ORs for distinct studies was 0.19 to 5.16. With a 95% confidence interval (CI) of [0.24, 4.41], the total OR came out to be 1.03. AF and mortality after GI surgeries were deemed inconsequential when the CI crossed the line of no effect (OR = 1), indicating that there was no significant difference in mortality between the experimental and control groups when looking at the combined effect estimate. Additionally, the association was deemed negligible since the *p*-value, which is larger than the 0.05 threshold, was found to be *p* = 0.97. The I^2^ value of 88% found during our analysis suggests significant heterogeneity across the included studies. This indicates that there was more to the differences in research results than just random variance. Therefore, there was no discernible difference in atrial fibrillation-related mortality between the experimental and control groups, according to this forest plot. However, careful interpretation is required due to the significant level of heterogeneity.

### 3.3. Atrial Fibrillation

**Figure 5:**
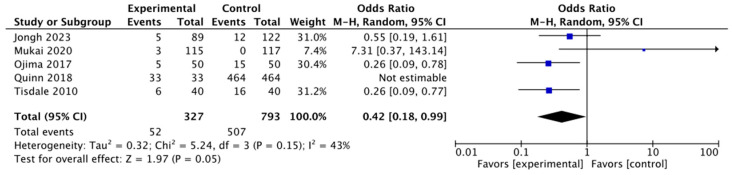
Atrial fibrillation forest plot [14,15,16,17,18].

For the second investigation, our variable was atrial fibrillation. We wanted to assess which group had highest weightage of AF. This forest plot included five studies: Jongh (2023) [14], Mukai (2020) [15], Ojima (2017) [16], Quinn (2018) [18] and Tisdale (2010) [17]. For each study, the forest plot showed the number of events (AF) in the experimental and control groups. For the experimental group, we took participants who underwent gastrointestinal surgical procedures in the selected studies, whereas for the control group, we took data from whichever control group was present in each study (Figure 5). Our idea was to see which group had more chance of developing atrial fibrillation that could subsequently lead to a higher chance of mortality. The total number of subjects in each group and the odds ratio of atrial fibrillation in the experimental group were compared to those of the control group. The total number of patients in the experimental group from the five studies was 327 patients, whereas in the control group, the total number of patients was 793. The forest plot showed a statistically significant association atrial fibrillation in the experimental group with a *p* value of *p* = 0.05. The overall odds ratio was 0.42 (95% confidence interval, 0.18 to 0.99). The analysis showed some evidence of heterogeneity between studies with I^2^ = 43%.

**Figure 6:**
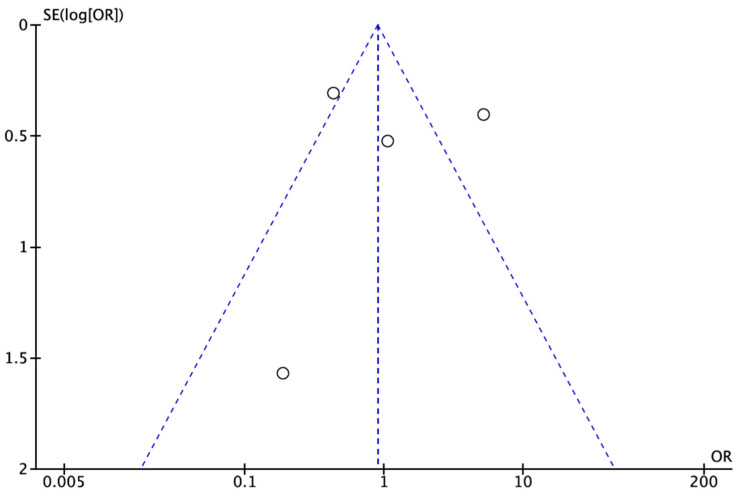
Funnel plot.

The results we obtained provide evidence that there is no significant correlation between mortality caused by atrial fibrillation and abdominal surgery. In addition, we created a funnel plot using the Revman program to evaluate the presence of publication bias and to provide a visual representation of it (Figure 6). 

## 4. Discussion

In order to better understand the connection between atrial fibrillation and mortality after gastrointestinal surgery, we conducted this systematic review and meta-analysis. Our results show that there is no evidence of a significant death from atrial fibrillation after gastrointestinal surgery (*p* = 0.97, not significant), and the combined OR was 1.03 with a 95% confidence interval (CI) of [0.24, 4.41]. Furthermore, we calculated that the experimental group had a higher incidence of atrial fibrillation than that of the control group. After major abdominal surgery, the fatality rate may be as high as 17%, but it is typically between 3% and 7%. However, it is important to mention the secondary causes of AF that could potentially result in death. Frost et al. mentioned in their study that there are other secondary causes of AF too, which are hyperthyroidism, cardiac disease and hypertension [19]. Furthermore, Mont et al. mentioned that regular and extreme endurance sport practice, atrial ectopic beats, inflammatory changes, atrial size, increased vagal tones, bradycardia, occupational physical activity, vigorous physical activity, structural atrial changes and long-lasting sport practice all potentially pose a risk for atrial fibrillation [20]. Moreover, alcohol consumption, smoking [21] and channelopathies [22] are other risk factors. Contradicting our findings, a cohort study by Rühlmann et al. indicated that atrial fibrillation is likely to be linked to higher mortality after upper gastrointestinal surgery. However, it is important to note that Rühlmann et al. also suggested a link between postoperative AF and higher mortality rates during hospital stays as well as prolonged stays in the ICU. This increased risk may be because of extended stays. Furthermore, their investigation demonstrated an independent relationship between in-hospital mortality and the incidence of new-onset postoperative AF [23]. In contrast to the earlier study, Cormack and associates reported in their research publication that 20% of patients had new-onset AF, a common postoperative outcome for esophageal and junctional carcinoma. They also found other risk factors for AF, such as diabetes mellitus, aging, pre-existing cardiac issues, radiation or chemotherapy given before surgery, pneumonia after surgery, accumulation of pleural fluid and elevated inflammatory markers. However, no association was found between AF and death, suggesting that AF did not worsen these patients’ prognoses [24]. In 2023, Rühlmann et al. also undertook a study with the aim of ascertaining whether postoperative atrial fibrillation reduces the risk of mortality and gastrointestinal surgery. In their research, they discovered a correlation by which lower intestinal tract surgeries increase the risk of atrial fibrillation, which may ultimately result in mortality [25]. The authors of an observational study provided support for the assertion that atrial fibrillation exhibits the most substantial correlation with 90-day mortality among patients who have undergone gastrointestinal surgery, as indicated by a hazard ratio of 4.4 (2.8–6.8). A total of 33 of the 63 patients at risk of developing atrial fibrillation in their study ultimately died. A total of 52% of fatalities were ascribed to atrial fibrillation. It was the highest number of patients to die as a result of complications associated with atrial fibrillation throughout the entire research study. A meta-analysis conducted by Ravi et al. assessed the mortality benefit of catheter ablation for AF versus medical therapy only, and the catheter ablation method turned out to be superior with a *p* value of 0.003 [26]. Furthermore, Senes et al. talked about the ablate and pace strategy with cardiac resynchronization therapy or pacing on conduction systems. The authors found that, among both groups, the active group had a shorter paced QRS width (128 ± 18 ms vs. 148 ± 27 ms, *p* = 0.004). The QRS was also shorter in the 12 patients who had HBP + LV (128 ± 13 ms, *p* = 0.02 vs. controls) than it was in the controls. Death, hospitalization for HF or worsened HF happened to three people (11%) in the active group and to four people (15%) in the control group over a mean of 9.7 and 9.5 months of follow up, respectively [27]. In addition to that, coronary calcium volume is a significant risk factor for mortality, with higher volumes associated with increased risk. Hypertensive patients with a coronary calcium score > 400 mm3 had the highest risk of hospital death [28].

Regarding observational studies, we came across a meta-analysis conducted by Albini 2021 et al. from Italy. After a thorough review of the literature, the researchers found eight papers that satisfied their inclusion requirements. These studies examined the incidence of stroke, death and recurrence of atrial fibrillation in individuals with POAF. When POAF developed, there was a four-fold long-term increase in the risk of stroke, according to the meta-analysis of the included studies. Furthermore, patients with POAF had a 3.5-fold greater risk of death than postoperative patients without POAF, according to an analysis of two trials that included long-term mortality data [29]. This meta-analysis contradicts our findings. However, it is worth noting here that this research study included an observational approach, whereas we preferred a clinical trial approach. Patients with POAF had greater recurrence rates, according to Albini and colleagues. However, an accurate study could not be conducted due to variations in recurrence detection techniques and follow-up periods. This topic must be studied in greater detail with different settings and study designs to better understand the reliability of the analysis from different trials and studies [29].

It is noteworthy that a higher proportion of patients undergoing lower gastrointestinal surgery were susceptible to atrial fibrillation, in comparison to patients undergoing upper gastrointestinal surgery [30]. It is speculated that the insignificance of the combined aggregating result of our analysis may have been attributable to the scarcity of available randomized trials. The heterogeneity present in the data warrants circumspect interpretation as well. 

## 5. Future Implications

Our work could have substantial impacts on clinical practice, research and healthcare policy in the future. This study brings important light to the link between atrial fibrillation and death after gut surgery. These data may be included in future clinical practice suggestions, which would help medical practitioners make well-informed choices about patient care. The results may also be used by doctors to improve prior risk evaluations for stomach surgery patients. Preoperative planning and resource distribution may be changed by the fact that there is no link between atrial fibrillation and postoperative death. Furthermore, this study stresses the need of conducting further research to explore the particular factors that lead to death after stomach surgery. Subsequent study tries may look into other probable effects or conditions that might possibly have a more notable influence on patient outcomes. Future studies should aim to include a bigger range of study subjects, taking into account different geographic areas, surgery methods and groups. This might improve the data’s generalizability and provide a fuller understanding of the topic in question. Finally, while making regulations related to stomach surgery, policymakers might take the study’s results into account. Healthcare systems’ decisions on how to divide resources and create policies may be affected by the very little effect of atrial fibrillation on death. 

## 6. Limitations

This study has some drawbacks and limitations as well. The authors would like to note that the potential presence of publication bias may affect the reliability of the results. Funnel plots were used to assess this bias, and there was publication bias evident in this study. Moreover, the studies included in the meta-analysis varied in terms of surgical procedures, study duration and patient populations, as shown in Table 1. This heterogeneity could impact the generalizability of the findings of this study. The problem that we faced the most was that the analysis was based on a relatively small number of studies (n = 5) due to not enough data being available on this topic, which might limit the statistical power and generalizability of the results. Furthermore, despite the strict inclusion and exclusion criteria, the possibility of inherent bias in the selection of studies could not be completely eliminated. This may impact the validity of the overall findings. One more limitation that we would like to highlight here is the inclusion criteria of the studies. We included studies that were published only in English, and this might have introduced language bias, potentially excluding relevant studies published in other languages. The exclusion of patients under 18 years old may limit the applicability of the findings to the broader population, as some surgical procedures may be performed on younger patients. The focus on mortality due to atrial fibrillation as the primary outcome may have overlooked other relevant outcomes or complications associated with gastrointestinal surgery and atrial fibrillation. Last, the findings may not be universally applicable, as they were derived from studies with specific inclusion criteria, potentially limiting their generalizability to diverse clinical settings. Hence, broader studies are required to tackle this issue.

## 7. Conclusions

In conclusion, this systematic review and meta-analysis, following PRISMA guidelines, investigated the link between atrial fibrillation and mortality after gastrointestinal surgery. Although this study found a limited association and insignificant results reflected by an odds ratio of 1.03, the heterogeneity among studies and potential publication bias warrant cautious interpretation. The findings of this study underscore the need for future research diversifying study populations and exploring intervention strategies. Despite its limitations, this study contributes valuable insights that can inform clinical practice guidelines and policy decisions, highlighting the ongoing pursuit of nuanced evidence in surgical outcomes.

## Figures and Tables

**Table 1:** Study characteristics.

DOI	Author	Study Type	Duration of Study	Surgery	Total Population	Experimental Population	Control Population	Mean Age Means/Sd. Deviation		Atrial Fibrillation		Mortality	
								Experimental	Control	Experimental	Control	Experimental	Control
10.1016/j.bja.2020.08.060	Jongh 2023 [14]	RCT	3 years	Distal and Total gastrectomy	211	89	122	N/A	N/A	5	12	7	9
10.1016/j.bja.2020.08.060	Mukai 2020 [15]	RCT	2 years	Transthoracic Oesophagectomy	232	115	117	4.2 ± 84	3.6 ± 83	3	0	22	41
10.1002/bjs.10548	Ojima 2017 [16]	RCT	3 years	Oesophagectomy	100	50	50	68 (31–85)	69 (45–83)	5	15	N/A	N/A
10.1016/j.jtcvs.2010.01.026	Tisdale 2010 [17]	RCT	4 years	Transthoracic Esophagectomy	80	40	40	61 ± 10	63 ± 9	6	16	0	2
10.1111/ans.14484	Quinn 2018 [18]	RCT	2 years	Colorectal surgery	497	33	464	73.51 ± 11.3	62.60 ± 15.6	33	464	11	41

N/A—not applicable.

## Data Availability

Not applicable.

## Abbreviations

AF	Atrial Fibrillation
GI	Gastrointestinal
RCT	Randomized Controlled Trial
PRISMA	Preferred Reporting Items for Systematic Reviews and Meta-Analyses
PICO	Population, Intervention, Comparator group and Outcomes
CI	Confidence Interval
OR	Odds Ratio
ICU	Intensive Care Unit
POAF	Postoperative Atrial Fibrillation
N/A	Not Applicable
Sd. Deviation	Standard Deviation
ICTRP	International Clinical Trials Registry Platform
CINAHL	Cumulative Index to Nursing and Allied Health Literature
MeSH	Medical Subject Headings
DOI	Digital Object Identifier
RevMan	Review Manager
